# Effect of Tea (*Camellia sinensis*) and Olive (*Olea europaea* L.) Leaves Extracts on Male Mice Exposed to Diazinon

**DOI:** 10.1155/2013/461415

**Published:** 2013-04-18

**Authors:** Atef M. Al-Attar, Isam M. Abu Zeid

**Affiliations:** Department of Biological Sciences, Faculty of Sciences, King Abdulaziz University, P.O. Box 139109, Jeddah 21323, Saudi Arabia

## Abstract

The present study was aimed to evaluate the effects of tea and olive leaves extracts and their combination in male mice intoxicated with a sublethal concentration of diazinon. Exposure of mice to 6.5 mg/kg body weight of diazinon for seven weeks resulted in statistical increases of serum alanine aminotransferase, aspartate aminotransferase, gamma glutamyl transferase, alkaline phosphatase, creatine kinase, creatinine, glucose, triglycerides, and cholesterol, while the value of serum total protein was declined. Treating diazinon-intoxicated mice with tea and olive leaves extracts or their combination significantly attenuated the severe alterations in these hematobiochemical parameters. Moreover, the results indicated that the supplementation with combination of tea and olive leaves extracts led to more attenuation effect against diazinon toxicity. Additionally, these new findings suggest that the effect of tea and olive leaves extracts and their combination against toxicity of diazinon may be due to antioxidant properties of their chemical constituents. Finally, the present study indicated that the extracts of tea and olive leaves and their combination can be considered as promising therapeutic agents against hepatotoxicity, cardiotoxicity, nephrotoxicity, and metabolic disorders induced by diazinon and maybe by other toxicants and pathogenic factors.

## 1. Introduction

Organophosphorus compounds are one of the most common types of organic pollutants found in the environment [1]. Diazinon (C_12_H_21_N_2_O_3_PS) is an organophosphorus insecticide which is widely used in agriculture, and to control pests in the environment, this compound can be highly toxic for animals and human kind [2, 3]. Diazinon affects mainly the nervous system regardless of the route of exposure. Toxic effects of diazinon are due to the inhibition of acetylcholinesterase activity, an enzyme needed for proper nervous system function [4]. Some mild signs and symptoms of poisoning include headache, dizziness, weakness, feelings of anxiety, constriction of the pupils, and blurred vision. More severe symptoms include nausea and vomiting, abdominal cramps, slow pulse, diarrhea, pinpoint pupils, difficulty breathing, coma, and possibly death. These effects also occur in animals exposed to high doses of diazinon. There is no evidence that long-term exposure to low levels of diazinon causes harmful effects in people. Diazinon has not been shown to affect fertility in humans and it has hazardous side effects on humans, and economic farm animals that accidentally exposed to it [5, 6]. Furthermore, several investigations have showed that diazinon was capable of inducing histopathological, biochemical, and physiological changes [7–9].

Recently, there has been worldwide interest in the role of medicinal plants in complementary medicine. Extracts prepared from medicinal plants and other natural sources contain a variety of molecules with potent biological activities [10]. Tea is one of the most widely consumed beverages in the world, second only to water, and its medicinal properties have been widely explored. The tea plant, *Camellia sinensis*, is a member of the Theaceae family, and black, oolong, and green tea are produced from its leaves. Tea contains polyphenols, especially green tea, which include flavanols, flavadiols, flavonoids, and phenolic acids that may account for up to 30% of the dry weight. Certain catechins are the most biologically active group of the polyphenols in tea components [11]. Much evidence indicates that tea polyphenols have various biological activities including antifungal, anti-inflammation, antimutagenic, antioxidative, anticarcinogenic, antitumor, antidiabetic effects, the lowering of plasma cholesterol and triglyceride levels, and reduction of blood pressure and platelet aggregation in several systems [12–15]. The olive tree (*Olea europaea *L.), family: Oleaceae, and, in particular, its leaves have been used for the treatment of wounds, fever, diabetes, gout, atherosclerosis, and hypertension since ancient times [16]. Olive leaf has been traditionally used for centuries to prevent and treat different diseases. It is used to enhance the immune system, in heart disease, and as an antimicrobial agent. Experimental animal studies on different total olive leaves extract or their constituents have demonstrated hypoglycemic, hypotensive, antiarrhythmic, antiatherosclerotic, vasodilator, antihepatotoxic, and antinephrotoxic effects [17–20]. Therefore, the aim of the present study was to investigate the effects of tea and olive leaves extracts and their combination on hematobiochemical alterations in mice exposed to a sublethal concentration of diazinon.

## 2. Materials and Methods

### 2.1. Experimental Animals

The experiments were done using male albino mice of MF1 strain, weighing 24–31 g. The mice were obtained from the Experimental Animal Unit of King Fahd Medical Research Center, King Abdulaziz University, Jeddah, Saudi Arabia. The principles of laboratory animal care were followed throughout the duration of experiment and the instruction given by King Abdulaziz University Ethical Committee was followed regarding experimental treatments. The experimental animals were housed in standard plastic cages and maintained under controlled laboratory conditions of humidity (65%), temperature (25 ± 1°C), and 12 : 12 h light: dark cycle, with balanced food and water ad libitum. 

### 2.2. Tea and Olive Leaves Extraction

Fresh young leaves of tea were directly collected from some tea plantation farms in Cameron Highlands, Malaysia. The leaves were thoroughly washed and dried at room temperature. The fine quality of dried leaves was kept in dry plastic container until being used for extract preparation. The dried tea leaves (10 g) were powdered and added to 500 mL cold water and mixed in an electric mixer for 10 minutes. Fine-quality olive leaves, 10% oleuropein (General Nutrition Corporation, Pittsburgh, USA), were used for the preparation of an aqueous extract. The olive leaves (10 g) were powdered and put in 500 mL cold water and mixed in an electric mixer for 10 minutes. Thereafter, the solutions of olive and tea leaves were filtered, and the filtrates were evaporated in an oven at 40°C to produce dried residues (active principles). With references to the powdered samples, the yields of the olive and tea extracts were 16.7% and 17.3%, respectively. Furthermore, these extracts were weekly prepared and stored in refrigerator for subsequent experiments.

### 2.3. Experimental Treatments

 A total of sixty-four mice were randomly divided into eight experimental groups eight mice each. The experimental groups were treated as follows.

1. Mice of group 1 were served as controls and intraperitoneally injected with saline solution (0.9% NaCl), five times weekly, for seven weeks.
2. Mice of group 2 were intraperitoneally given diazinon at the level of 6.5 mg/kg body weight (1/10 of LD_50_), five times weekly, for seven weeks.
3. Animals of group 3 were orally supplemented with tea leaves extract (400 mg/kg body weight) and after four hours received diazinon at the same dose given to group 2, five times weekly, for seven weeks.
4. Mice of group 4 were orally supplemented with olive leaves extract (400 mg/kg body weight) and after four hours received diazinon at the same dose given to group 2, five times weekly, for seven weeks.
5. Animals of group 5 were orally supplemented with olive leaves extract (200 mg/kg body weight) and tea leaves extract (200 mg/kg body weight) and after four hours received diazinon at the same dose given to group 2, five times weekly, for seven weeks.
6. Mice of group 6 were intraperitoneally received saline solution at the same dose given to group 1 and were orally supplemented with tea leaves extract at the same dose given to group 3, five times weekly, for seven weeks.
7. Mice of group 7 were intraperitoneally received saline solution at the same dose given to group 1 and were orally supplemented with olive leaves extract at the same dose given to group 4, five times weekly, for seven weeks.
8. Animals of group 8 were intraperitoneally received saline solution at the same dose given to group 1 and were supplemented with tea and olive leaves extracts at the same dose given to group 5, five times weekly, for seven weeks.

### 2.4. Hematobiochemical Analyses

After seven weeks, mice were anaesthetized with diethyl ether. Blood was collected from orbital venous plexus in nonheparinized tubes and centrifuged at 2000 rpm for 20 minutes, and blood sera were then collected and stored at 4°C till the determination time of alanine aminotransferase (ALT), aspartate aminotransferase (AST), gamma glutamyl transferase (GGT), alkaline phosphatase (ALP), creatine kinase (CK), creatinine, glucose, total protein, triglycerides, and cholesterol using Automated Clinical Chemistry Analysis System, Dimension type RXL Max (Dade Behring Delaware, DE 19714, USA) and automatic analyzer (Reflotron Plus System, Roche, Germany).

### 2.5. Statistical Analysis

Data were expressed as the mean ± standard deviation (SD) and were analyzed by one-way analysis of variance (ANOVA) using Statistical Package for Social Sciences (SPSS for windows, version 12.0). Multiple comparative analyses were conducted between all experimental groups using Tukey's test. Results were considered statistically significant at *P* < 0.05. 

## 3. Results

Significant increases in the values of serum ALT (+181.2%), AST (+188.1%), GGT (+364.4%), ALP (+153.3%), CK (+112.9%), creatinine (+396.8%), glucose (+99.8%), triglycerides (+82.4%), and cholesterol (+135.0%) were observed in mice exposed to diazinon (group 2) when compared to control mice and other treated groups (Tables 1 and 2). Moreover, the value of serum total protein (−15.3%) was statistically declined in mice of group 2 when compared to control mice and other treated groups (Table 2). In comparison with control mice, the values of serum ALT, AST, GGT, ALP, CK, creatinine, glucose, and cholesterol were statistically increased in mice treated with tea leaves extract plus diazinon (group 3), olive leaves extract plus diazinon (group 4), and tea and olive leaves extracts plus diazinon (group 5). Insignificant alterations of serum total protein were observed in mice of groups 3, 4, and 5. The value of serum triglycerides was statistically increased in mice treated with tea leaves extract plus diazinon (group 3), olive leaves extract plus diazinon (group 4), while the value of serum triglycerides was significantly unchanged in mice supplemented with tea and olive leaves extracts plus diazinon (group 5). Additionally, no statistically significant differences were detected in the values of all hematobiochemical parameters in mice treated with tea leaves extract (group 6), olive leaves extract (group 7), and tea and olive leaves extracts (group 8) except the value of serum cholesterol in mice treated with olive leaves extract (group 8) which declined compared with control group. Furthermore, Tables 1 and 2 demonstrate that the highly percentage changes of the studied hematobiochemical parameters in mice subjected to diazinon were reduced by treatments of tea leaves extract (group 3), olive leaves extract (group 4), and by the combination of tea and olive leaves extracts (group 5). Collectively, the results indicate that the supplementation with combination of tea and olive leaves extracts (group 5) led to more attenuation effect against diazinon toxicity.

## 4. Discussion

This is the first study on the effects of tea and olive leaves extracts and their combination on toxicity of diazinon in male mice. As seen in the present study, the extracts of tea and olive leaves were investigated for antitoxicity of diazinon in male mice. The values of serum ALT, AST, GGT, ALP, CK, creatinine, glucose, triglycerides, and cholesterol were significantly higher, while the value of serum total protein was statistically declined in mice exposed to only diazinon. These findings are mostly in agreement with different previous studies which indicated that the administration of diazinon and other pesticides caused severe physiological and biochemical alterations in experimental animals [7–9, 20–22]. The observed increase in the levels of ALT, AST, GGT, and ALP is the major diagnostic symptoms of liver dysfunctions, and these enzymes have been used as markers for monitoring chemically induced tissue damages [23]. Furthermore, several investigators showed that these enzymes liberate to the blood stream when the hepatic parenchyma cells are damaged in experimental animals exposed to diazinon and other pesticides [8, 20–22]. 

The present increase of serum CK value in mice exposed to diazinon may be due to the damage and necrosis of cardiac muscle tissues. CK is the first heart enzyme to appear in the blood after a heart attack, and it also disappears quickly from the blood. However, several researchers reported that the exposure to diazinon and other pesticides led to cardiotoxicity in experimental animals [8, 9, 24]. The present study shows that mice exposed to diazinon display a pronounced impairment of renal function which is conformed by the enhancement of serum creatinine. Furthermore, a disorder of kidney function reduces excretion of creatinine, resulting in increased blood creatinine levels. Thus, creatinine levels give an approximation of the glomerular filtration rate. It is however known that the increase of creatinine occurred with renal failure [25]. The obtained results showed that diazinon intoxication caused increases in the values of serum glucose, triglycerides, and cholesterol, while the value of serum total protein was statistically declined. These results indicate that the exposure to diazinon caused a severe disturbance of carbohydrates, lipids, and proteins metabolism. The progressive accumulation of blood glucose revealed that mice became hyperglycemic due to diazinon intoxication. This case may be due to the enhancement of the activities of the enzymes involved in gluconeogenesis leading to formation of glucose from noncarbohydrate sources coupled with the inhibition of liver glycogenolysis or stimulating glycogenolysis processes to increase the level of blood glucose from liver as a main source of carbohydrates in the body. Concerning the present hypertriglyceridemia of diazinon-treated mice, a number of hepatotoxic agents also cause accumulation of fatty deposits, predominantly triglycerides in the parenchyma cells in the liver. Agbor et al. [26] reported that the accumulation of blood triglycerides may be as a result of an imbalance between the rate of synthesis and the rate of release of triglycerides by the parenchyma cells into the systemic circulation. The present work showed an enhancement of serum cholesterol, hypercholesterolemia, in mice exposed to diazinon. Generally, the pesticides inhibit hepatic cytochrome P-450 enzymes. The increase in cholesterol level indicates inhibitory action of pesticide on cytochrome P-450 enzymes. Moreover, increased cholesterol concentration indicates liver disorders and cholestasis [27, 28]. Total serum protein, the majority of serum proteins which are synthesized in the liver, is used as an indicator of liver impairment [29]. The present hypoproteinemia could be due to a decrease in the rate of protein synthesis and/or due to several pathological processes including liver injury, renal damage, and elimination of protein in the urine [30, 31]. 

The present results indicated that treating diazinon-intoxicated mice with tea and olive leaves extracts or their combination significantly attenuated the severe alterations in hematobiochemical parameters. These observations were confirmed by the decline of percentage changes of the studied parameters. Moreover, the combination of tea and olive leaves extracts exerts more attenuation influence against diazinon toxicity. It has been previously suggested that several pesticides exert their biological effects mainly through electrophilic attack of cellular constituents with simultaneous generation of reactive oxygen species (ROS). ROS may, therefore, be involved in the toxicity of various pesticides [32]. Pesticide chemicals may induce oxidative stress leading to the generation of free radicals and alteration in antioxidants or oxygen free radical (OFR) scavenging enzyme systems [33]. It is important to note that many environmental contaminants, such as pesticides, accumulate in fatty tissues [34]. Tissue degeneration is a free-radical mediated process that involves lipid peroxides and lipid peroxidation of polyunsaturated fatty acids (PUFAs) of the mammalian tissue [35]. Therefore, lipid peroxidation has been suggested as one of the molecular mechanisms involved in pesticide-induced toxicity [33]. Furthermore, many authors postulate that the organophosphorus compounds may have an effect on redox processes in a number of organs, thus leading to disturbances in these processes and causing enhancement of lipid peroxidation, both in acute and chronic intoxication by these compounds [36, 37]. The traditional tea (*Camellia sinensis*) infusion is characterized by a high content of flavonoids. Flavonoids are a large group of phenolic products of plant metabolism with a variety of phenolic structures that have unique biological properties and may be responsible for many of the health benefits attributed to tea. Tea is an important source of flavonoids in the diet, and the flavonoids found in tea are known to be strong antioxidants. Many in vitro studies show that the flavonoids present in tea have strong antioxidant and metal-chelating properties and may therefore protect cells and tissues against free oxygen radicals [38]. El-Kott and Bin-Meferij [39] reported that the supplementation of crude extract of green tea repaired and recovered of lung tissue injuries induced by malathion, an organophosphorus pesticide, in rats. They suggested that this effect may be due to the antioxidant, antitoxin, and chemoprotective properties of different components of green tea. Korany and Ezzat [40] compared between green tea and black seed, *Nigella sativa*, extracts in the prevention of fenitrothion-induced toxicity on rat parotid salivary gland. They concluded that the administration of natural antioxidants could be of beneficial effect on the prevention of cytotoxicity induced by organophosphorous compounds and green tea showed more promising results than that of black seed. Additionally, Zari and Al-Attar [20] showed that the pretreatment with olive leaves extract improved the hematological, biochemical, and histopathological alterations of liver, kidney, and testis induced by carbendazim, a fungicide, intoxication in male mice. This indicated the effectiveness of olive leaves extract in the prevention of carbendazim toxicity. Furthermore, they suggested that the olive leaves extract exerts its ameliorative effect against carbendazim-induced hematological, biochemical, and histopathological alterations by preventing the decline of antioxidant defense system and direct free radical scavenging activity. In conclusion, the present study showed that the pretreatment of tea and olive leaves extracts and their combination attenuates the severe hematobiochemical alterations induced by diazinon intoxication. Finally, we suggest that the effects of tea and olive leaves extracts and their combination against hepatotoxicity, cardiotoxicity, nephrotoxicity, and metabolic disorders were induced by diazinon, possibly due to antioxidant properties of their natural chemical constituents. Further physiological, biochemical and histopathological investigations are needed to explore the possible use of different doses of these crude extracts and their constituents as potential natural preventive agents against the influences of diazinon and may be against other toxicants and pathogenic factors.

## Figures and Tables

**Table 1:** Concentration of ALT, AST, GGT, ALP, and CK in serum from control, diazinon, tea leaves extract plus diazinon, olive leaves extract plus diazinon, tea and olive leaves extracts plus diazinon, tea leaves extract, olive leaves extract, and tea and olive leaves extracts treated mice after seven weeks. Percentage changes are included in parentheses.

Treatments	Parameters				
	ALT (U/L)	AST (U/L)	GGT (U/L)	ALP (U/L)	CK (U/L)
Control	26.67 ± 1.86	36.33 ± 2.16	5.42 ± 9.76	101.00 ± 8.32	306.50 ± 8.17
Diazinon	75.00 ± 8.37^ab^ (+181.2)	104.67 ± 18.10^ab^ (+188.1)	25.17 ± 3.36^ab^ (+364.4)	255.83 ± 46.94^ab^ (+153.3)	652.50 ± 35.14^ab^ (+112.9)
Tea leaves + diazinon	45.50 ± 1.65^a^ (+70.6)	68.67 ± 8.19^a^ (+89.0)	11.67 ± 2.34^a^ (+115.3)	153.33 ± 20.63^a^ (+51.8)	420.00 ± 55.05^a^ (+37.0)
Olive leaves + diazinon	46.33 ± 2.42^a^ (+73.7)	55.00 ± 12.28^a^ (+51.4)	14.12 ± 3.55^a^ (+160.5)	159.30 ± 29.60^a^ (+57.7)	456.67 ± 69.22^a^ (+49.0)
Tea and olive leaves + diazinon	36.50 ± 5.01^a^ (+36.9)	45.80 ± 8.52^a^ (+26.1)	8.00 ± 1.79^a^ (+47.6)	142.67 ± 23.76^a^ (+41.3)	419.00 ± 83.67^a^ (+36.7)
Tea leaves	25.50 ± 2.74 (−4.4)	37.66 ± 3.72 (+3.7)	5.16 ± 0.82 (−4.8)	102.66 ± 11.30 (+1.6)	311.33 ± 24.15 (+1.8)
Olive leaves	26.00 ± 2.68 (−2.5)	36.70 ± 2.38 (+1.0)	5.25 ± 0.52 (−3.1)	97.00 ± 9.01 (−4.0)	308.83 ± 20.05 (+0.8)
Tea and olive leaves	25.33 ± 2.34 (−5.0)	36.42 ± 2.88 (+0.3)	5.20 ± 0.68 (−4.1)	100.50 ± 10.78 (−0.4)	299.33 ± 15.36 (−2.3)

Data represent the means ± SD of 6 animals per group. ^a^Indicates a significant difference between control and treated groups. ^b^Indicates a significant difference between mice exposed to diazinon and tea leaves extract plus diazinon, olive leaves extract plus diazinon, tea and olive leaves extracts plus diazinon, tea leaves extract, olive leaves extract, and tea and olive leaves extracts treated mice.

**Table 2:** Concentration of creatinine, glucose, total protein, triglycerides, and cholesterol in serum from control, diazinon, tea leaves extract plus diazinon, olive leaves extract plus diazinon, tea and oil leaves extracts plus diazinon, tea leaves extract, olive leaves extract, and tea and olive leaves extracts treated mice after seven weeks. Percentage changes are included in parentheses.

Treatments	Parameters				
	Creatinine (mg/dL)	Glucose (mg/dL)	Total protein (g/dL)	Triglycerides (mg/dL)	Cholesterol (mg/dL)
Control	0.63 ± 0.14	88.17 ± 11.30	5.63 ± 0.43	83.33 ± 5.65	94.83 ± 5.11
Diazinon	3.13 ± 1.40^ab^ (+396.8)	176.17 ± 48.44^a^ (+99.8)	4.77 ± 0.36^ab^ (−15.3)	152.00 ± 31.51^ab^ (+82.4)	222.80 ± 31.61^ab^ (+135.0)
Tea leaves + diazinon	1.60 ± 0.92^a^ (+154.0)	132.33 ± 20.30^a^ (+50.0)	5.25 ± 0.24 (−6.8)	103.67 ± 15.06^a^ (+24.4)	163.17 ± 36.99^a^ (+72.1)
Olive leaves + diazinon	1.53 ± 0.33^a^ (+142.9)	125.50 ± 15.22^a^ (+42.3)	5.42 ± 0.23 (−3.7)	98.50 ± 16.83^a^ (+18.2)	141.33 ± 23.71^a^ (+49.0)
Tea and olive leaves + diazinon	1.48 ± 0.51^a^ (+134.9)	160.67 ± 26.55^a^ (+82.2)	5.38 ± 0.31 (−4.4)	82.00 ± 5.93 (−1.6)	128.66 ± 8.36^a^ (+35.6)
Tea leaves	0.70 ± 0.14 (+11.1)	93.17 ± 20.23 (+5.7)	5.58 ± 0.22 (−0.9)	80.00 ± 4.56 (−4.0)	94.00 ± 7.72 (−0.9)
Olive leaves	0.53 ± 0.18 (−15.9)	75.83 ± 7.94 (−14.0)	5.53 ± 0.35 (−1.8)	79.83 ± 4.12 (−4.2)	82.67 ± 6.95^a^ (−12.8)
Tea and olive leaves	0.67 ± 0.16 (+6.4)	87.83 ± 0.87 (−0.4)	5.35 ± 0.34 (−5.0)	80.17 ± 7.49 (−3.8)	92.16 ± 11.22 (−2.8)

Data represent the means ± SD of 6 animals per group. ^a^Indicates a significant difference between control and treated groups. ^b^Indicates a significant difference between mice exposed to diazinon and tea leaves extract plus diazinon, olive leaves extract plus diazinon, tea and olive leaves extracts plus diazinon, tea leaves extract, olive leaves extract, and tea and olive leaves extracts treated mice.
